# Supplementary material for: Sex differences in the reward value of familiar mates in prairie voles
Source: Genes Brain Behav. 2022 Jan 19;21(3):e12790. doi: 10.1111/gbb.12790 (PMC8917082; doi:10.1111/gbb.12790)
Supplement: Supplementary file 1 — Figure S1 Castration and hormone capsule implantation of male stimulus animals did not influence female lever pressing responses for access. Females housed with a male partner were tested on sequential days with intact strangers and strangers who were castrated and implanted with testosterone capsules designed to reproduce T levels in the physiological range (Costantini et al., 2007). Females exhibited no apparent differences in lever pressing effort towards males of either type, with no significant difference between groups in a pairwise t‐test, and a near‐zero mean of differences (error bars represent 95% confidence interval). [file GBB-21-e12790-s001.pdf]

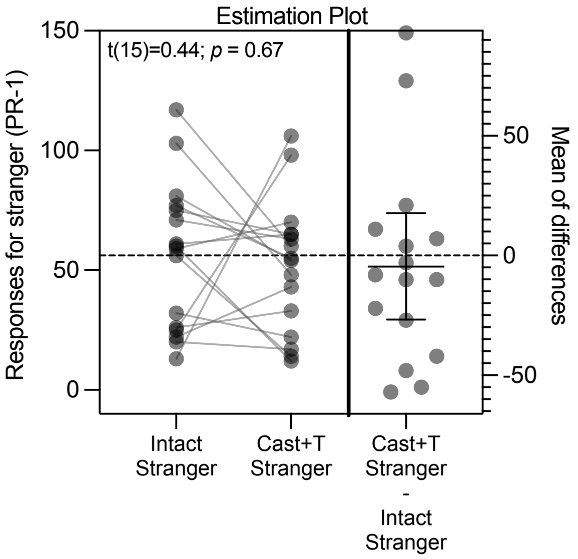

Figure S1. Castration and hormone capsule implantation of male stimulus animals did not influence female lever pressing responses for access. Females housed with a male partner were tested on sequential days with intact strangers and strangers who were castrated and implanted with testosterone capsules designed to reproduce T levels in the physiological range (Costantini et al. 2007). Females exhibited no apparent differences in lever pressing effort towards males of either type, with no significant difference between groups in a pairwise t-test, and a near-zero mean of differences (error bars represent 95% confidence interval).
